# Co-transduction of dual-adeno-associated virus vectors in the neonatal and adult mouse utricles

**DOI:** 10.3389/fnmol.2022.1020803

**Published:** 2022-10-19

**Authors:** Zhong-Rui Chen, Jing-Ying Guo, Lu He, Shan Liu, Jun-Yi Xu, Zi-Jing Yang, Wei Su, Ke Liu, Shu-Sheng Gong, Guo-Peng Wang

**Affiliations:** ^1^Department of Otolaryngology-Head and Neck Surgery, Beijing Friendship Hospital, Capital Medical University, Beijing, China; ^2^Clinical Center for Hearing Loss, Capital Medical University, Beijing, China

**Keywords:** adeno-associated virus, gene transfer, utricle, mice, hair cell, transduction

## Abstract

Adeno-associated virus (AAV)-mediated gene transfer is an efficient method of gene over-expression in the vestibular end organs. However, AAV has limited usefulness for delivering a large gene, or multiple genes, due to its small packaging capacity (< 5 kb). Co-transduction of dual-AAV vectors can be used to increase the packaging capacity for gene delivery to various organs and tissues. However, its usefulness has not been well validated in the vestibular sensory epithelium. In the present study, we characterized the co-transduction of dual-AAV vectors in mouse utricles following inoculation of two AAV-serotype inner ear (AAV-ie) vectors via canalostomy. Firstly, co-transduction efficiencies were compared between dual-AAV-ie vectors using two different promoters: cytomegalovirus (CMV) and CMV early enhancer/chicken β-actin (CAG). In the group of dual AAV-ie-CAG vectors, the co-transduction rates for striolar hair cells (HCs), extrastriolar HCs, striolar supporting cells (SCs), and extrastriolar SCs were 23.14 ± 2.25%, 27.05 ± 2.10%, 57.65 ± 7.21%, and 60.33 ± 5.69%, respectively. The co-transduction rates in the group of dual AAV-ie-CMV vectors were comparable to those in the dual AAV-ie-CAG group. Next, we examined the co-transduction of dual-AAV-ie-CAG vectors in the utricles of neonatal mice and damaged adult mice. In the neonatal mice, co-transduction rates were 52.88 ± 3.11% and 44.93 ± 2.06% in the striolar and extrastriolar HCs, respectively, which were significantly higher than those in adult mice. In the *Pou4f3^+/DTR^* mice, following diphtheria toxin administration, which eliminated most HCs and spared the SCs, the co-transduction rate of SCs was not significantly different to that of normal utricles. Transgene expression persisted for up to 3 months in the adult mice. Furthermore, sequential administration of two AAV-ie-CAG vectors at an interval of 1 week resulted in a higher co-transduction rate in HCs than concurrent delivery. The auditory brainstem responses and swim tests did not reveal any disruption of auditory or vestibular function after co-transduction with dual-AAV-ie vectors. In conclusion, dual-AAV-ie vectors allow efficient co-transduction in the vestibular sensory epithelium and facilitate the delivery of large or multiple genes for vestibular gene therapy.

## Introduction

Peripheral vestibular dysfunction is a significant cause of imbalance and dizziness. Lesions of the sensory epithelium of vestibular end organs, such as Meniere’s disease (Tsuji et al., 2000b), aminoglycoside ototoxicity (Tsuji et al., 2000a), and syndromic inherited diseases (Jones and Jones, 2014), are common causes of peripheral vestibular dysfunction. Currently, drugs and the vestibular rehabilitation are clinically available treatments for those patients; However, the functional recovery is largely insufficient in some cases, particularly in those of bilateral vestibular hypofunction (Chow et al., 2021).

Gene therapy is a promising strategy for functional recovery and repair of the inner ear sensory epithelium. Adeno-associated virus (AAV) is a commonly used gene transfer vector and proved safe and effective in clinical trials (Nathwani et al., 2011; Yla-Herttuala, 2012; MacLaren et al., 2014). AAV is lowly immunogenic, non-integrating, and efficient in transducing dividing and non-dividing cells (Kay, 2011). Furthermore, single local administration is suitable for long-term treatment (Kay, 2011). However, AAV has limited packaging capacity (<5 kb), which limits its clinical usefulness (Reisinger, 2020). Gene therapy for certain inner ear diseases requires delivery of target genes larger than 5 kb, such as *Myo VIIa* for Usher syndrome type 1B and *Otof* for DFNB9 (Jones and Jones, 2014). Recent studies have reported that the delivery of a single therapeutic gene is inadequate to induce functional HC regeneration. Manipulation of multiple transcription factors is required for HC regeneration and functional recovery of the inner ear (Costa et al., 2015; Wu et al., 2016; You et al., 2018; Menendez et al., 2020). Therefore, increasing the packaging capacity of AAV would enable the delivery of large or multiple genes, thereby increasing the clinical usefulness of the system.

Substantial efforts have been made to circumvent the packaging limit of AAV vectors (Reisinger, 2020). Several studies have attempted to express protein fragments using AAV vectors loaded with truncated cDNAs, to provide partial gene function (Liu et al., 2005; Ostedgaard et al., 2005). In the inner ear of zebrafish, C-terminal C2F domain of otoferlin, but not the N-terminal C2A domain, can restore hearing and balance (Chatterjee et al., 2015). However, AAV-mediated expression of otoferlin fragments in mammals failed to improve hearing in an otoferlin knockout (Otof-/-) model (Tertrais et al., 2019). Thus, protein fragments differ in terms of their capacity to induce functional recovery. This heterogeneity is a major barrier to the application of protein fragments.

Based on the intrinsic ability of AAV genomes to achieve intermolecular concatemerization (Duan et al., 1998), co-transduction of dual-AAVs in a single cell has been used. Different strategies are used to split a large gene expression cassette into halves, which are independently packaged in two AAV vectors (Trapani et al., 2014). Co-transduction by dual-AAV vectors partially rescued the auditory function in otoferlin knockout mice and *Tmc*1 mutant mice (Akil et al., 2019; Al-Moyed et al., 2019; Wu et al., 2021). This method rescued the vestibular function after co-injection of dual-AAV vectors into a neonatal mouse model of Usher syndrome type 1c (Pan et al., 2017). However, it is not clear whether co-transduction of dual-AAV vectors is effective for the vestibular end organs of normal or damaged adult mice. It is also unknown whether sequential administration of dual-AAV vectors leads to favorable co-transduction, which is necessary for the overexpression of multiple genes in a sequential manner. Furthermore, the long-term performance of dual-AAV vectors needs to be explored.

In this study, we evaluated the efficiency and safety of dual-AAV vectors for the vestibular end organs of mice. For this purpose, we used AAV-serotype inner ear (AAV-ie), which is an efficient vector for inner ear gene transfer (Tan et al., 2019). We assessed the co-transduction efficiency of dual-AAV-ie vectors after concurrent or sequential administration under different circumstances, including normal and damaged adult and neonatal mouse utricles, which has not yet been explored in detail. We found that dual-AAV-ie vectors provided satisfactory co-transduction efficiency with minimum damage to the inner ear.

## Materials and methods

### Adeno-associated virus vectors

We used purified AAV-ie viral vectors driven by the promoters of cytomegalovirus (CMV) or CMV early enhancer/chicken β-actin (CAG). AAV-ie with reporter genes of enhanced green fluorescent protein (EGFP) or mCherry were used for the experiments. The vectors were purchased from PackGene Biotech Co., Ltd. (Guangzhou, Guangdong, China) at a titer of 1 × 10^13^ vg/mL. The vectors were generated by triple plasmid transfection into HEK293T cells. The titers of the vectors were determined using Droplet Digital PCR. Vector aliquots were stored in phosphate-buffered saline (PBS) with 0.001% pluronic F-68 at -80°C.

### Animals and diphtheria toxin treatments

The animal experiments were conducted according to the guidelines of the Animal Care and Use Committee of Capital Medical University of China. Wild-type C57BL/6J (6–8-week-old) and CD-1 (postnatal day 1, P1) mice were purchased from SPF Biotechnology Co., Ltd. (Beijing, China). *Pou4f3^+/DTR^* mice were purchased from the Jackson Laboratory (Bar Harbor, ME, US) and bred in the Laboratory Animal Department at Capital Medical University of China. *Pou4f3^+/DTR^* mice (8–10 weeks old) received two intramuscular injections of 100 ng/g DT (List Biological Laboratories, Campbell, CA, USA) 1 day apart. The surgeries were performed 10 days later.

### Surgeries

Adult mice were anesthetized via intraperitoneal injection of xylazine (7 mg/kg; Sigma-Aldrich, St Louis, MO, USA) and ketamine (120 mg/kg; Gutian Pharmaceutical Co., Gutian, Fujian, China). Ketoprofen (10 mg/kg; Sigma-Aldrich) was subcutaneously injected immediately before the operation. Sedation was induced and maintained in neonatal mice using hypothermic anesthesia. The surgeries were performed only on the left ears. After shaving and sterilization of the overlying skin, an incision was made in the left post-auricular region. The posterior or lateral semicircular canal was exposed after separation of the overlying muscles. The surgeries were performed as previously described (Wang et al., 2014; Guo et al., 2017, 2018). For concurrent injection of dual-AAV-ie vectors, two AAV-ie vectors (1 μl for each) were mixed prior to the surgery. Mixed vector suspensions were inoculated via the posterior semicircular canal at a rate of 0.5 μl/min using a micro-injection pump. For the sequential administration of dual vectors, 1 μl of AAV-ie-CAG-EGFP vector was injected through the lateral semicircular canal, followed 1 week later by a second injection of 1 μl of AAV-ie-CAG-mCherry through the posterior semicircular canal. Animals were euthanized 2 weeks or 3 months after the surgery.

### Auditory brainstem response and swim tests

ABR and swim tests were performed 2 weeks or 3 months after the surgery. For the ABR test, anesthetized animals were placed on a heating mat in an electrically and acoustically shielded chamber. Subdermal needle electrodes were placed at the vertex (active), and beneath the pinna of the test ear (reference) and contralateral ear (ground). Acoustic stimuli (5-ms tone bursts) were generated by the Tucker Davis Technologies (TDT) System III hardware and SigGenRZ software (TDT, Alachua, FL, USA). The responses evoked at octave frequencies of 4, 8, 16, and 32 kHz were recorded. A total of 1,024 responses were averaged for each stimulus level at 5-dB intervals. The threshold was defined as the lowest stimulus level at which ABR waves could be reliably detected. To evaluate vestibular function, the swim tests were performed as previously described (Hardisty-Hughes et al., 2010).

### Immunofluorescence staining

Following deep anesthesia and euthanasia, the temporal bones of the mice were harvested and fixed in 4% paraformaldehyde and PBS overnight at 4°C. After rinsing with PBS, utricles were collected, permeabilized in 0.3% Triton X-100 (Sigma-Aldrich) for 20 min, and blocked with 5% normal goat serum (ZSGB-BIO, Beijing, China) for 1 h at room temperature. The samples were incubated overnight at 4°C with the following primary antibodies: rabbit anti-myosin VIIa antibody (1:300; Proteus BioSciences Inc., Ramona, CA, USA), mouse anti-GFP antibody tagged with Alexa Fluor 488 (1:100; Santa Cruz Biotechnology Inc., Dallas, TX, USA), and rat anti-mCherry antibody (1:100; Invitrogen, Carlsbad, CA, USA). After rinsing with PBS, samples were incubated with fluorescence-labeled secondary antibodies tagged with Alexa Fluor 594 or 647 (1:300; Invitrogen) for 1 h at room temperature. The nuclei were stained with 4’,6-diamidino-2-phenylindole (DAPI; 1:1,000; AppliChem, Darmstadt, Germany) for 5 min at room temperature. After rinsing with PBS, samples were mounted on glass slides with Fluoromount-G (Southern Biotech, Birmingham, AL, USA). Confocal images were obtained using a scanning confocal microscope (TCS SP8, Leica Camera AG, Wetzlar, Germany). Pictures were cropped, labeled, and spaced using WPS Office software (Kingsoft Office Software, Inc., Beijing, China).

### Cell counts and co-transduction efficiency analysis

The cells were counted using confocal images obtained with a 63 × objective lens and an additional 2 × digital zoom. The images were processed using ImageJ software (National Institutes of Health, Bethesda, MD, USA). Three views of the striolar and extrastriolar regions each (∼90 × 90 μm per view) were randomly captured at the level of the cuticular plate of HCs for HC counting, and at the level of supporting cell (SC) nuclei for SC counting. At the level of the cuticular plate of HCs, the numbers of GFP-positive/mCherry-positive/myosin VIIa-positive cells (co-transduced HCs) and all myosin VIIa-positive cells (HCs) were recorded and divided to obtain the co-transduction rate of HCs. Similarly, at the level of SC nuclei, the numbers of GFP-positive/mCherry-positive cells (co-transduced SCs) and all DAPI-positive cells (SCs) were recorded and divided to obtain the co-transduction rate of SCs. Each group included 5–6 samples.

### Statistical analyses

Data are presented as mean ± standard error of mean (SEM). Statistical analyses were performed using GraphPad Prism 9 software (GraphPad Software, Inc., San Diego, CA, USA). Statistical differences in co-transduction efficiency were determined using Student’s *t*-test. Differences were considered statistically significant when the *P*-value was < 0.05.

## Results

### Co-transduction of dual-adeno-associated virus-ie vectors was efficient in normal adult mouse utricle

The transduction profile of dual-AAV-ie was investigated using AAV-ie vectors driven by CAG or CMV promoters. Dual-AAV-ie vector mixtures, AAV-ie-CAG-EGFP and AAV-ie-CAG-mCherry, or AAV-ie-CMV-EGFP and AAV-ie-CMV-mCherry, were, respectively, inoculated into the adult mouse inner ear. The utricles were sampled 2 weeks after the surgery.

**FIGURE 1:**
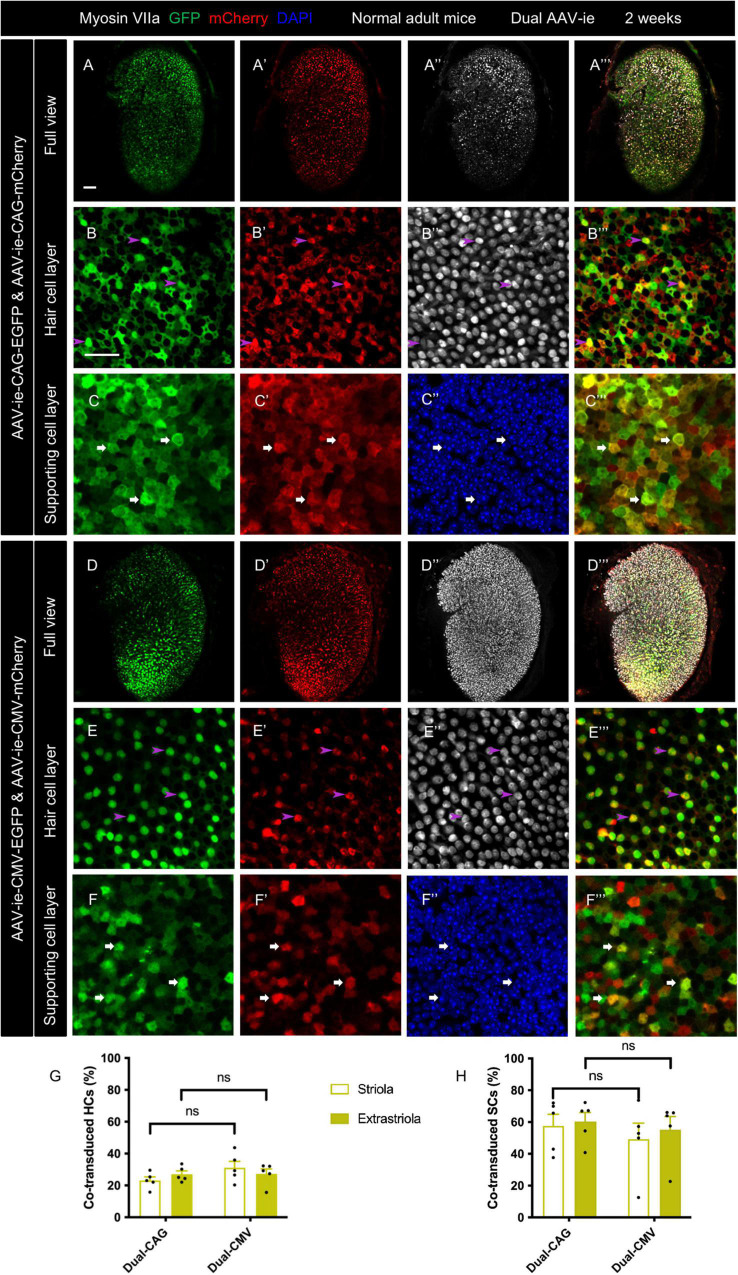
Co-transduction of dual-AAV-ie vectors in the normal adult mouse utricle. Dual AAV-ie-CAG vectors (AAV-ie-CAG-EGFP and AAV-ie-CAG-mCherry) or dual AAV-ie-CMV vectors (AAV-ie-CMV-EGFP and AAV-ie-CMV-mCherry) were inoculated into the inner ear of adult mice. Utricles were harvested 2 weeks after the surgery. **(A–C”’)** Low- **(A–A”’)** and high- **(B–C”’)** magnification images show extensive co-expression of GFP and mCherry in both HCs (arrowheads in **B–B”’**; representative images of the extrastriolar region) and SCs (arrows in **C–C”’**; representative images of the striolar region) after transduction by dual AAV-ie-CAG vectors. **(D–F”’)** Abundant HCs (arrowheads in **E–E”’**; representative images of the extrastriolar region) and SCs (arrows in **F–F”’**; representative images of the extrastriolar region) express both GFP and mCherry after transduction by dual-AAV-ie-CMV vectors. Scale bars, 50 μm in A for **(A–A”’)** and **(D–D”’)**; 20 μm in B for the remaining images. **(G,H)** Quantitative analysis showing that dual-AAV-ie-CAG and dual-AAV-ie-CMV vectors achieve comparable co-transduction rates in HCs **(G)** and SCs **(H)**. Data are mean ± SEM. *P*-values were calculated by Student’s *t*-test. “ns”, not significant.

The whole mounts of utricles revealed robust GFP and mCherry expression throughout the utricles after dual-AAV-ie transduction in both groups (Figures 1A–F”’). Co-localization of GFP and mCherry was extensively found in the vestibular HCs and SCs of both dual-AAV-ie groups, indicating co-transduction by dual-AAV-ie vectors. The HCs stained by the myosin VIIa antibody exhibited minimal morphological damage after dual-AAV-ie transduction.

The co-transduction rates of HCs and SCs from the striolar and extrastriolar regions were quantitatively analyzed (Table 1). The co-transduction rates of HCs (Figure 1G) and SCs (Figure 1H) were not significantly different between the dual-AAV-ie-CAG and dual-AAV-ie-CMV groups (striolar HCs: 23.14 ± 2.25% *vs.* 31.14 ± 4.02%, *P* = 0.1206; extrastriolar HCs: 27.05 ± 2.10% *vs.* 27.31 ± 3.05%, *P* = 0.9454; striolar SCs: 57.65 ± 7.21% *vs.* 49.27 ± 10.05%, *P* = 0.5171; extrastriolar SCs: 60.33 ± 5.69% *vs.* 55.17 ± 8.29%, *P* = 0.6217, respectively). There was no significant difference in co-transduction rates of HCs or SCs between the striolar and the extrastriolar regions in each dual-AAV-ie group.

**TABLE 1:** Summary of the co-transduction rate of each group.

Groups	Striolar HCs%	Extrastriolar HCs%	Striolar SCs%	Extrastriolar SCs%
Dual AAV-CAG-2w (*N* = 5)	23.14 ± 2.25	27.05 ± 2.10	57.65 ± 7.21	60.33 ± 5.69
Dual AAV-CMV-2w (*N* = 5)	31.14 ± 4.02	27.31 ± 3.05	49.27 ± 10.05	55.17 ± 8.29
Neonatal-2w (*N* = 5)	52.88 ± 3.11	44.93 ± 2.06	71.92 ± 5.47	72.11 ± 3.61
Damaged-2w (*N* = 6)	56.78 ± 8.15	59.28 ± 7.81	66.18 ± 6.02	65.11 ± 6.45
Adult-3m (*N* = 5)	27.96 ± 4.32	33.11 ± 1.40	64.96 ± 5.13	74.71 ± 3.64
Neonatal-3m (*N* = 5)	35.60 ± 5.60	31.43 ± 4.38	44.41 ± 8.72	45.68 ± 9.32
Damaged-3m (*N* = 5)	36.13 ± 3.60	39.15 ± 4.03	66.57 ± 4.52	65.88 ± 4.36
Sequential-2w (*N* = 6)	38.87 ± 1.25	37.59 ± 0.55	75.46 ± 2.99	71.96 ± 3.51

### Dual adeno-associated virus-ie vectors allowed co-transduction in the normal neonatal mouse utricle and damaged adult mouse utricle

**FIGURE 2:**
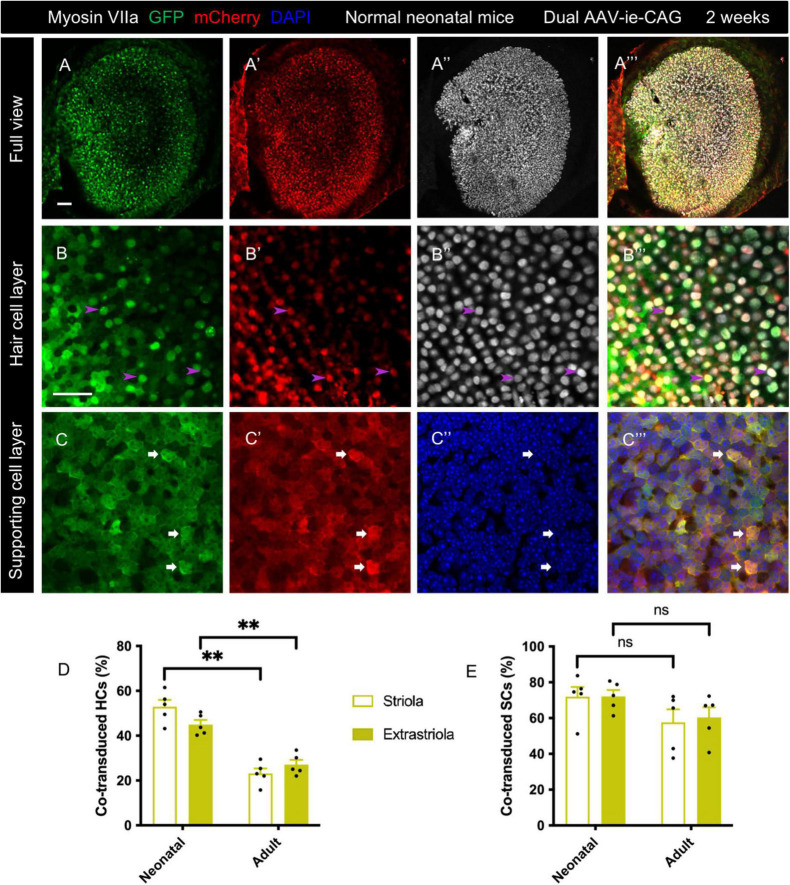
Co-transduction of dual AAV-ie vectors in the normal utricle of neonatal mice. AAV-ie-CAG-EGFP and AAV-ie-CAG-mCherry were injected into the neonatal mice at postnatal day 1 (P1). The utricles were sampled at P15. **(A–A”’)** Low-magnification images. **(B–C”’)** Numerous hair cells (HCs) (**B–B”’**; arrowheads; representative images of the extrastriolar region) and supporting cells (SCs) (**C–C”’**; arrows; representative images of the striolar region) express both GFP and mCherry. Scale bars, 50 μm in A for **(A–A”’)**; 20 μm in B for **(B–C”’)**. **(D,E)** Comparative analysis of the co-transduction rates of HCs **(D)** and SCs **(F)** between normal neonatal and adult mice. The co-transduction rates of HCs in neonatal mice are significantly higher than in adult mice in striolar and extrastriolar regions, whereas the co-transduction rates of SCs are comparable between the groups. Data are mean ± SEM. *P*-values were calculated using Student’s *t*-test. “ns”, not significant. **P < 0.01.

The transduction profiles of dual-AAV-ie were assessed in normal neonatal mice. A mixture of AAV-ie-CAG-EGFP and AAV-ie-CAG-mCherry vectors was injected at P1. The co-transductions were evaluated 2 weeks later. Intense co-expression of GFP and mCherry was seen throughout the sensory epithelium (Figure 2) and transitional epithelium of the utricle (Supplementary Figure 1). The co-transduction rates were 52.88 ± 3.11%, 44.93 ± 2.06%, 71.92 ± 5.47%, and 72.11 ± 3.61% in striolar HCs, extrastriolar HCs, striolar SCs, and extrastriolar SCs, respectively (Table 1). Compared to the co-transduction rate of dual AAV-ie-CAG vectors in normal adult mice (Table 1), that of HCs in neonatal mice was significantly higher in both the striolar and extrastriolar regions (Figure 2D; striolar HCs: 52.88 ± 3.11% *vs.* 23.14 ± 2.25%, *P* < 0.01; extrastriolar HCs: 44.93 ± 2.06% *vs.* 27.05 ± 2.10%, *P* < 0.01, respectively), although the co-transduction rates of SCs were comparable (Figure 2E; striolar SCs: 71.92 ± 5.47% *vs.* 57.65 ± 7.21%, *P* = 0.1535; extrastriolar SCs: 72.11 ± 3.61% *vs.* 60.33 ± 5.69%, *P* = 0.1190, respectively).

**FIGURE 3:**
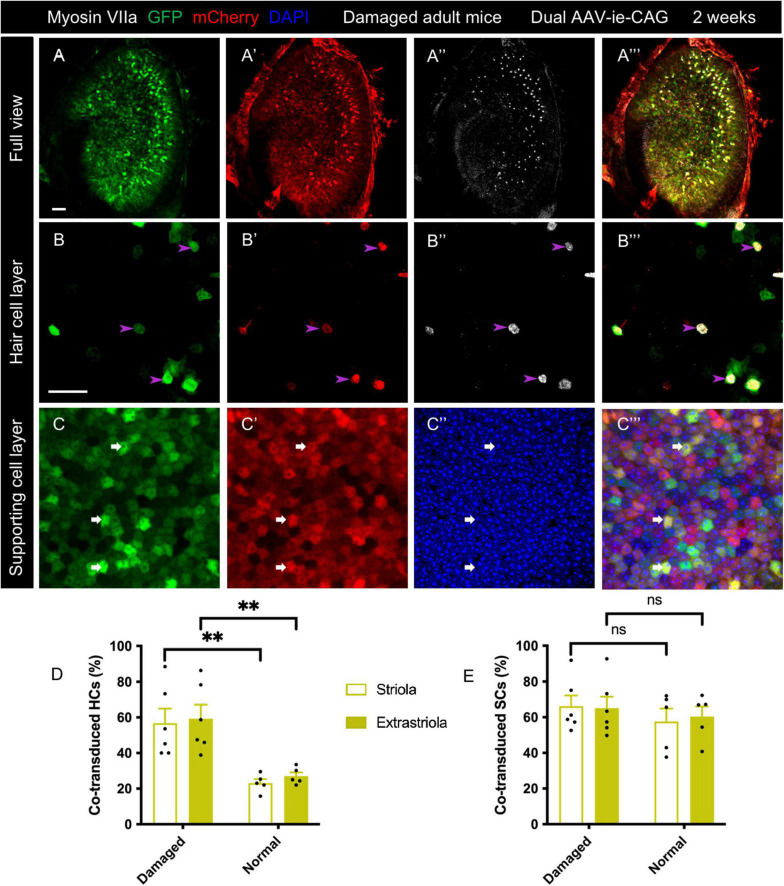
Co-transduction of dual-AAV-ie vectors in the damaged adult mouse utricle. AAV-ie-CAG-EGFP and AAV-ie-CAG-mCherry were injected into the inner ear of adult *Pou4f3^+/DTR^* mice 10 days following diphtheria toxin administration. Utricles were sampled 2 weeks after the surgery. **(A–A”’)** Low-magnification images show extensive expression of GFP and mCherry throughout the utricle, with the loss of most hair cells (HCs). **(B–C”’)** High-magnification images show co-transduction of dual-AAV-ie-CAG vectors in residual HCs (**B–B”’**; arrowheads; representative images of the extrastriolar region) and abundant supporting cells (SCs) (**C–C”’**; arrows; representative images of the extrastriolar region). Scale bars, 50 μm in A for **(A–A”’)**; 20 μm in B for **(B–C”’)**. **(D,E)** Comparative analysis of the co-transduction rates of HCs **(D)** and SCs **(E)** in damaged and normal adult mice. The co-transduction rates of HCs in damaged adult mice are significantly higher than those of normal mice in striolar and extrastriolar regions, whereas the co-transduction rates of SCs are comparable. Data are mean ± SEM. *P*-values were calculated using Student’s *t*-test. “ns,” not significant. **P < 0.01.

In the damaged group, most utricular HCs were experimentally ablated by two intramuscular injections of DT in adult *Pou4f3^+/DTR^* mice. The mixture of AAV-ie-CAG-EGFP and AAV-ie-CAG-mCherry vectors was injected 10 days after DT administration. Immunofluorescence staining of utricles was performed 2 weeks later. As indicated by the myosin VIIa staining, scattered HCs were present in the extrastriolar region of the utricle (Figures 3A–A”’). Intense over-expression of GFP and mCherry was present in HCs (Figures 3B–B”’) and SCs (Figures 3C–C”’). As shown in Table 1, the co-transduction rates of HCs in the striolar and extrastriolar regions were significantly higher than normal adult mice (Figure 3D; striolar HCs: 56.78 ± 8.15% *vs.* 23.14 ± 2.25%, *P* < 0.01; extrastriolar HCs: 59.28 ± 7.81% *vs.* 27.05 ± 2.10%, *P* < 0.01, respectively). No significant difference was observed in the co-transduction rate of SCs between the damaged and normal utricles in the striolar and extrastriolar regions (Figure 3E; striolar SCs: 66.18 ± 6.02% *vs.* 57.65 ± 7.21%, *P* = 0.3832; extrastriolar SCs: 65.11 ± 6.45% *vs.* 60.33 ± 5.69%, *P* = 0.6000, respectively). In addition, no significant difference was found in co-transduction rates of HCs or SCs between the striolar and the extrastriolar regions in neonatal or *Pou4f3^+/DTR^* mouse utricle.

### Co-transduction of dual-adeno-associated virus-ie vectors was maintained for up to 3 months in adult mice

**FIGURE 4:**
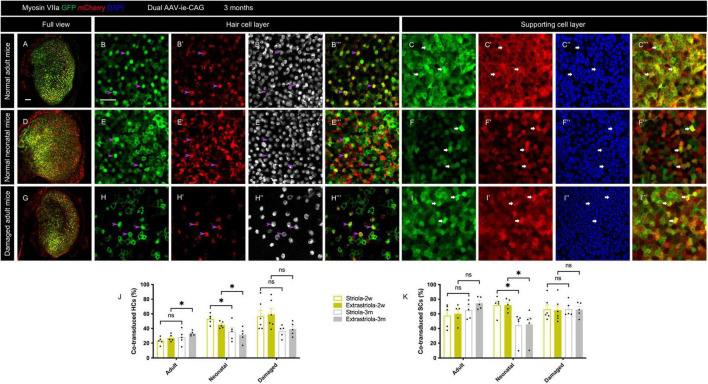
Long-term co-transduction of dual-AAV-ie vectors in the mouse utricle. Utricles were sampled 3 months after the injection of AAV-ie-CAG-EGFP and AAV-ie-CAG-mCherry. **(A–C”’)** Normal adult mice (**B–B”’** representative images of the extrastriolar region; (**C–C”’** representative images of the striolar region). **(D–F”’)** Normal neonatal mice (**E–E”’** representative images of the extrastriolar region; **F–F”’** representative images of the extrastriolar region). **(G–I”’)** Adult *Pou4f3^+/DTR^* mice following diphtheria toxin administration (**H-H”’**, representative images of the extrastriolar region; **I–I”’** representative images of the extrastriolar region). Scale bars, 50 μm in A for A, D and G; 20 μm in B for the remaining images. **(J,K)** Comparative analysis of the co-transduction rates of hair cells (HCs) **(J)** and supporting cells (SCs) **(K)** at 2 weeks and 3 months after dual-AAV-ie injection. In the normal adult mice, the co-transduction rate of extrastriolar HCs slightly increases at 3 months, while the co-transduction rates of striolar HCs and both regions of SCs are not significantly different at 3 months than 2 weeks. In the normal neonatal mice, the co-transduction rates of HCs and SCs decreases at 3 months. In addition, the co-transduction rates of HCs and SCs in the damaged adult mice are not significantly different at 3 months than 2 weeks. Data are mean ± SEM. *P*-values were calculated using Student’s *t*-test. “ns,” not significant. *P < 0.05.

To assess long-term transduction by dual-AAV-ie vectors, co-transduction efficiency was evaluated at 3 months after delivery of AAV-ie-CAG-EGFP and AAV-ie-CAG-mCherry to normal adult mice, normal neonatal mice, and damaged adult mice. Immunofluorescence staining revealed extensive and robust co-expression of GFP and mCherry in the utricular sensory epithelium of all three groups (Figures 4A–I”’). In normal adult mice, the co-transduction rate in striolar HCs showed no significant difference at 3 months and 2 weeks, but it was slightly increased in extrastriolar HCs at 3 months (striolar HCs: 27.96 ± 4.32% *vs.* 23.14 ± 2.25%, *P* = 0.3509; extrastriolar HCs: 33.11 ± 1.40% *vs.* 27.05 ± 2.10%, *P* <0.05, respectively). The co-transduction rates of SCs were comparable at 3 months and 2 weeks (striolar SCs: 64.96 ± 5.13% *vs.* 57.65 ± 7.21%, *P* = 0.4325; extrastriolar SCs: 74.71 ± 3.64% *vs.* 60.33 ± 5.69%, *P* = 0.0661, respectively).

The co-transduction rates in the normal neonatal mice were lower at 3 months than 2 weeks in both HCs and SCs (Figures 4J,K; striolar HCs: 35.60 ± 5.60% *vs.* 52.88 ± 3.11%, *P* < 0.05; extrastriolar HCs: 31.43 ± 4.38% *vs.* 44.93 ± 2.06%, *P* < 0.05; striolar SCs: 44.41 ± 8.72% *vs.* 71.92 ± 5.47%, *P* < 0.05; extrastriolar SCs: 45.68 ± 9.32% *vs.* 72.11 ± 3.61%, *P* < 0.05, respectively).

The damaged adult mice exhibited no significant difference in co-transduction rates between 3 months and 2 weeks (striolar HCs: 36.13 ± 3.60% *vs.* 56.78 ± 8.15%, *P* = 0.0594; extrastriolar HCs: 39.15 ± 4.03% *vs.* 59.28 ± 7.81%, *P* = 0.0601; striolar SCs: 66.57 ± 4.52% *vs.* 66.18 ± 6.02%, *P* = 0.9620; extrastriolar SCs: 65.88 ± 4.36% *vs.* 65.11 ± 6.45%, *P* = 0.9267, respectively).

### Sequential delivery of dual-adeno-associated virus-ie vectors resulted in a higher co-transduction rate in HCs than concurrent delivery

**FIGURE 5:**
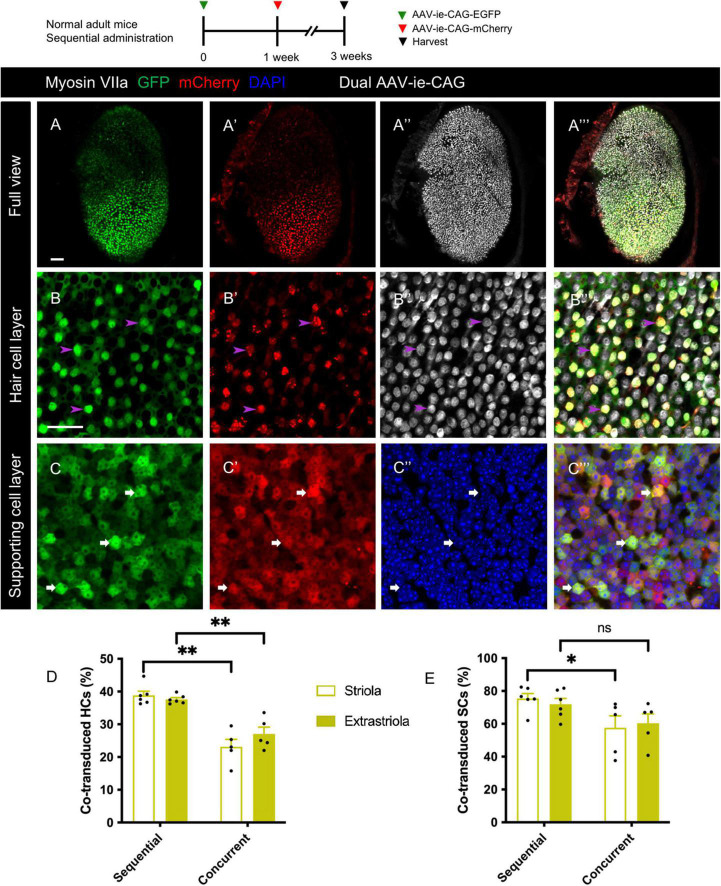
Co-transduction of dual-AAV-ie vectors in the normal adult mouse utricle after sequential administration. AAV-ie-CAG-EGFP was injected through the lateral semicircular canal, and AAV-ie-CAG-mCherry was injected through the posterior semicircular canal after 1 week. Utricles were sampled 2 weeks after the second injection. **(A–A”’)** Low-magnification images show extensive GFP and mCherry expression throughout the utricle. **(B–C”’)** Co-localization of GFP and mCherry expression is determined at the level of the cuticular plate of hair cells (HCs) (**B–B”’**; arrowheads; representative images of the striolar region) and the layer of supporting cell (SC) nuclei (**C–C”’**; arrows; representative images of the striolar region). Scale bars, 50 μm in A for **(A–A”’)**; 20 μm in B for **(B–C”’)**. **(D,E)** Comparative analysis of the co-transduction rates of HCs **(D)** and SCs **(E)** after sequential and concurrent injections. The co-transduction rates of striolar and extrastriolar HCs in the sequential group are significantly higher than those of the concurrent injection group. The co-transduction rate of striolar SCs show higher than that of the concurrent injection group, whereas the co-transduction rates of extrastriolar SCs is comparable between the groups. Data are mean ± SEM. *P*-values were calculated using Student’s *t*-test. “ns,” not significant. *P < 0.05, **P < 0.01.

Sequential delivery of dual vectors is sometimes required for the sequential over-expression of target genes in the sensory epithelium (Yao et al., 2018). After sequential administration of dual-AAV-ie-CAG vectors with an interval of 1 week, extensive co-expression of GFP and mCherry was found throughout the sensory epithelium (Figures 5A–C”’). The co-transduction rates of HCs after sequential administration of dual AAV-ie-CAG vectors were significantly higher than those after the concurrent injection of dual AAV-ie-CAG vectors (Figure 5D and Table 1; striolar HCs: 38.87 ± 1.25% *vs.* 23.14 ± 2.25%, *P* < 0.01; extrastriolar HCs: 37.59 ± 0.55% *vs.* 27.05 ± 2.10%, *P* < 0.01, respectively). The co-transduction rate of striolar SCs was higher after sequential administration than concurrent injection (Figure 5E and Table 1; 75.46 ± 2.99% *vs.* 57.65 ± 7.21%, respectively, *P* < 0.05), whereas the co-transduction rate of extrastriolar SCs after sequential administration was comparable to that after concurrent delivery (Figure 5E and Table 1; 71.96 ± 3.51% *vs.* 60.33 ± 5.69%, respectively, *P* = 0.1048).

### Concurrent delivery of dual-adeno-associated virus-ie vectors had minimal impact on the auditory and vestibular functions

**FIGURE 6:**
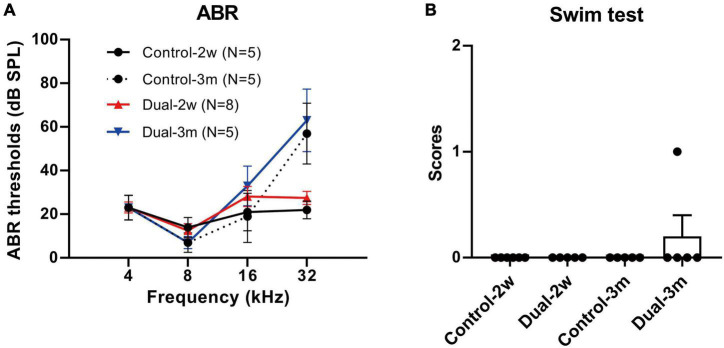
ABR thresholds and swim test scores 2 weeks and 3 months after co-transduction of dual-AAV-ie vectors in normal adult mice. **(A)** There is no significant difference in ABR thresholds between mice 2 weeks or 3 months after the injection of dual-AAV-ie vectors and control animals (i.e., age-matched mice without surgery) according to Student’s *t*-test at each frequency. **(B)** Swim test scores show no significant difference between the groups according to Student’s *t*-test. Data are mean ± SEM.

ABR and swim tests were performed 2 weeks and 3 months after the concurrent delivery of AAV-ie-CAG-EGFP and AAV-ie-CAG-mCherry in normal adult mice. Age-matched mice of the same background served as normal controls. As shown in Figure 6, no significant difference was found between the groups in terms of the ABR thresholds at 4, 8, 16, and 32 kHz frequencies, or the swim test scores, demonstrating that co-transduction of dual-AAV-ie vectors had minimal impact on the auditory and vestibular functions.

## Discussion

Dual-AAV-ie vectors achieved efficient co-transduction in the normal and damaged vestibular sensory epithelium of mice. The transduction was maintained for up to 3 months after co-transduction in adult mice. Sequential administration of dual-AAV-ie vectors was associated with a higher co-transduction rate in HCs than after concurrent delivery. Moreover, ABR and swim tests showed that co-transduction by dual-AAV-ie vectors minimally affected the inner ear function of normal mice. Taken together, the results showed that co-transduction by dual-AAV-ie vectors served as an efficient and safe approach for gene delivery to the mouse vestibular end organs.

Co-transduction of AAV vectors has been used *in vivo* in various tissues, such as the retina and cochlea (Colella et al., 2014; Carvalho et al., 2017; Al-Moyed et al., 2019; Omichi et al., 2020; Wu et al., 2021). Dual-AAV6 vectors allowed otoferlin overexpression in 19–30% of inner HCs of deaf Otof-/- mice and improved the deafness (Al-Moyed et al., 2019). Perinatal injection of a mixture of AAV-Anc80L65-harmonin-a1 and AAV-Anc80L65-harmonin-b1 improved deafness and vestibular dysfunction in Ush1c mice (Pan et al., 2017), demonstrating a possible role of dual-AAV method for gene delivery to the vestibular system. In the current study, we explored the possible applications of dual-AAV for normal and damaged vestibular end organs of adult mice. We demonstrated that sequential administration of dual-AAV vectors allows efficient co-transduction. The performance of dual-AAV vectors persisted for up to 3 months. Our results should aid the future application of dual-AAV vectors in the vestibular system.

Co-transduction efficiency might be affected by several processes. First, cellular entry largely depends on the multi-step interaction of viral capsids with receptors on the targeted cells (Zengel and Carette, 2020). However, the expression of AAV receptors in the mouse utricle has not been explored. Our data revealed that the co-transduction of dual-AAV-ie was significantly lower than that of single-AAV-ie injection (Tan et al., 2019). It might be because different age of mice and injection approach were used in Tan’s study, or due to the receptor competition when dual AAV-ie vectors were injected. Second, the intracellular events underlying the endomembranous cross and nuclear translocation remain largely unknown (Zengel and Carette, 2020). Finally, interaction between the promoters and RNA polymerase II (Domenger and Grimm, 2019) is important for transcription initiation. In the present study, dual-AAV-ie-CAG vectors and dual-AAV-ie-CMV vectors had comparable efficiency. Nevertheless, the cellular process of dual-AAV vectors remains unknown.

The postnatal stage provides a significant opportunity for the treatment of certain inherited inner ear diseases (Al-Moyed et al., 2019; Guo J. et al., 2021). Otoferlin overexpression at the end of the first postnatal week in the cochlea of Otof-/- mice is too late to prevent synapse degeneration (Al-Moyed et al., 2019). The capability of programmed cell cycle reactivity of the mouse inner ear declines sharply after birth (White et al., 2006), implying that gene therapy based on cell cycle manipulation should target the perinatal period. The present study showed that dual-AAV-ie-CAG was capable of efficient co-transduction in the utricle of neonatal mice (Figure 2), suggesting its potential usefulness for the aforementioned purposes. The co-transduction efficiencies of HCs and SCs were reduced at 3 months compared to 2 weeks (Figure 4), which might be explained by the active mitosis and differentiation of the utricular sensory epithelium during the neonatal period (Burns et al., 2012).

Genetic manipulation is a promising technique for HC regeneration and functional recruitment of damaged vestibular sensory epithelium (Li et al., 2016; Zhang et al., 2020); therefore, it has received significant attention. However, the induced HCs are insufficient in number, and in terms of maturation, which leads to variable functional outcomes (Schlecker et al., 2011; Guo J. Y. et al., 2021). There is a consensus that a single factor might not lead to sufficient regeneration of mature HCs (Shibata et al., 2020). Certain strategies have been used to manipulate multiple transcription factors or signaling pathways, and have achieved superior HC regeneration in transgenic mouse models (Costa et al., 2015; Kuo et al., 2015; Menendez et al., 2020; Chen et al., 2021; Iyer and Groves, 2021). However, transgenic mice cannot be used for clinical treatments; as an alternative, dual-AAV vectors with multiple target genes may be used. Therefore, the tropism of dual-AAV-ie-CAG vectors was tested herein in the damaged utricles of mice with experimental depletion of most HCs. The results showed that the co-transduction had higher efficiency in residual HCs, while it remained equivalent to normal mice in SCs, implying that it would be an efficient way to simultaneously overexpress multiple genes for HC regeneration.

Sufficient HC regeneration in the lesioned vestibular sensory epithelium may be achieved using a two-step reprogramming method: proliferation of SCs is stimulated, followed by the manipulation of essential transcription factors in SCs. Therefore, sequential over-expression of two target genes may be required. Sequential administration of dual-AAV vectors with different genes has been used for retinal diseases and successfully restored the vision of mice with congenital blindness (Yao et al., 2018), suggesting that this strategy might induce regeneration of the inner ear. Our data showed that sequential administration of dual-AAV-ie vectors resulted in satisfactory co-transduction in the mouse utricle (Figure 5), suggesting its potential usefulness for HC regeneration of vestibular sensory epithelium.

In summary, we comprehensively evaluated the co-transduction efficiency of dual-AAV vectors in the vestibular sensory epithelium under various conditions. Although the present study did not include therapeutic genes, understanding the co-transduction characteristics and safety profile of dual-AAV vectors may aid the delivery of large or multiple genes for vestibular gene therapy in the future.

## Data availability statement

The raw data supporting the conclusions of this article will be made available by the authors, upon reasonable request.

## Ethics statement

The animal study was reviewed and approved by Animal Care and Use Committee of Capital Medical University of China.

## Author contributions

Z-RC contributed to the conceptualization and methodology of the study and wrote the original draft. J-YG contributed to the conceptualization and methodology of the study, manuscript writing, reviewing, and editing. LH completed the data curation. SL, J-YX, and Z-JY conducted the surgeries. WS and KL were responsible for the software and validation. G-PW and S-SG provided the conceptualization, writing, reviewing, and supervision. All authors contributed to the article and approved the submitted version.

## Supplementary material

Supplementary Figure 1 Co-transduction of dual-AAV-ie vectors in the transitional epithelium of the neonatal mouse utricle. AAV-ie-CAG-EGFP and AAV-ie-CAG-mCherry were injected at postnatal day 1. The utricles were sampled 2 weeks **(A–B”)** or 3 months **(C–D”)** following injection. Robust co-expression of GFP (arrows in **B–B”**) and mCherry (arrows in **D–D”**) are present in the transitional epithelium. The dashed lines **(B”,D”)** delineate the boundary of the sensory and transitional epithelium. Scale bars, 50 μm in A for **(A,C)**; 10 μm in B for the remaining images.
