# Supplementary figures and images for: Co-transduction of dual-adeno-associated virus vectors in the neonatal and adult mouse utricles
Source: Front Mol Neurosci. 2022 Oct 19;15:1020803. doi: 10.3389/fnmol.2022.1020803 (PMC9629838; doi:10.3389/fnmol.2022.1020803)

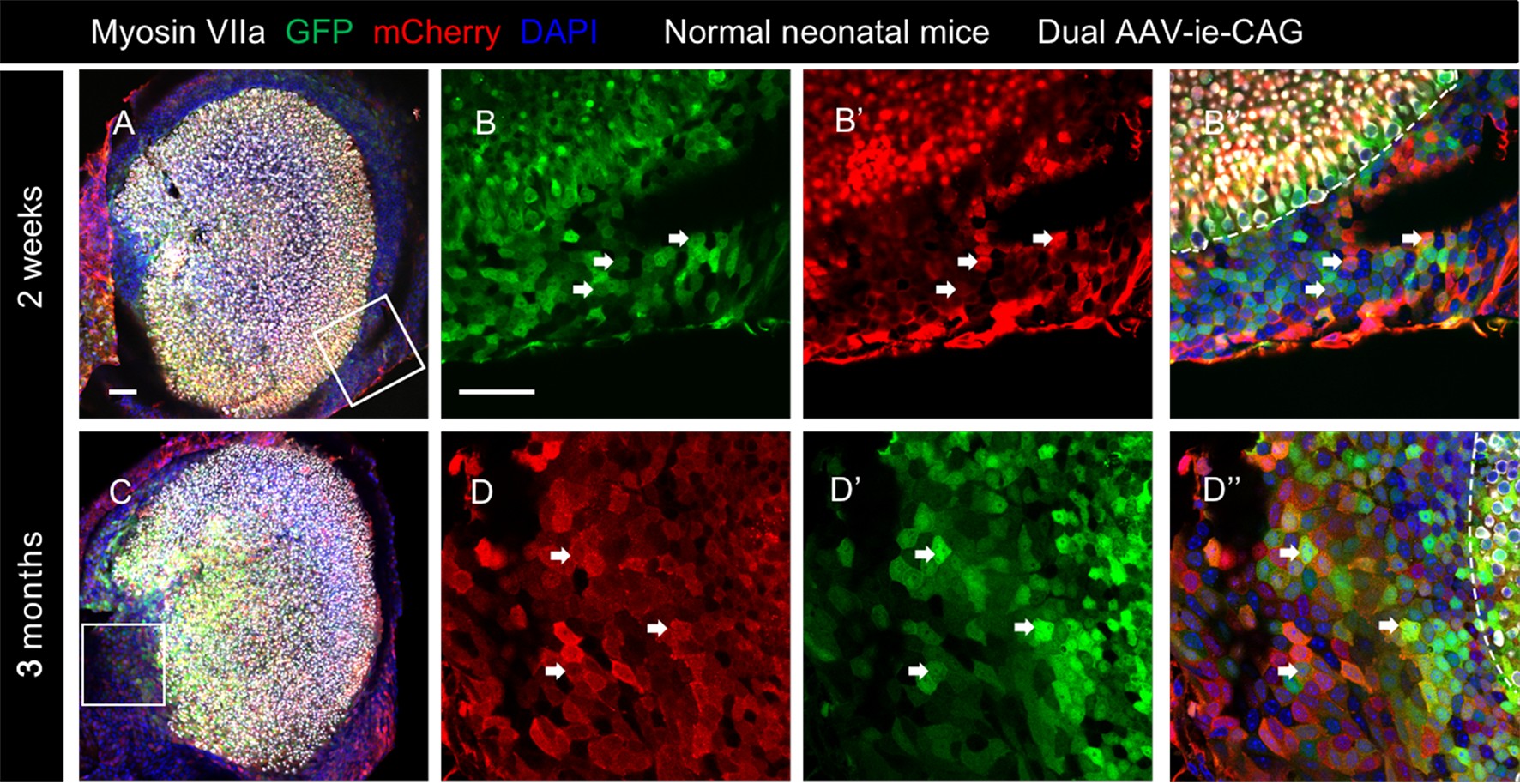

Supplement: Supplementary Figure 1 — Co-transduction of dual-AAV-ie vectors in the transitional epithelium of the neonatal mouse utricle. AAV-ie-CAG-EGFP and AAV-ie-CAG-mCherry were injected at postnatal day 1. The utricles were sampled 2 weeks (A–B”) or 3 months (C–D”) following injection. Robust co-expression of GFP (arrows in B–B”) and mCherry (arrows in D–D”) are present in the transitional epithelium. The dashed lines (B”,D”) delineate the boundary of the sensory and transitional epithelium. Scale bars, 50 μm in A for (A,C); 10 μm in B for the remaining images. [file Image_1.TIF]
